# Leaf Caloric Value from Tropical to Cold-Temperate Forests: Latitudinal Patterns and Linkage to Productivity

**DOI:** 10.1371/journal.pone.0157935

**Published:** 2016-06-24

**Authors:** Guangyan Song, Jihua Hou, Ying Li, Jiahui Zhang, Nianpeng He

**Affiliations:** 1 Key Laboratory for Forest Resources & Ecosystem Processes of Beijing, Beijing Forestry University, Beijing, 100083, China; 2 Key Laboratory of Ecosystem Network Observation and Modeling, Institute of Geographic Sciences and Natural Resources Research, Chinese Academy of Sciences, Beijing, 100101, China; DOE Pacific Northwest National Laboratory, UNITED STATES

## Abstract

Leaf caloric value (LCV) reflects the capacity of a leaf to fix and accumulate solar energy through photosynthesis. We systematically investigated the LCV of 745 plant species in 9 forests, representing a range of tropical to cold-temperate forests along the 4700-km North-South Transect of Eastern China. The goals were to explore the latitudinal patterns of LCV at the levels of species, plant functional group, and community and to establish the relationship between LCV and gross primary productivity (GPP). Our results showed that LCV for all species ranged from 12.85 to 22.15 KJ g^–1^ with an average of 18.46 KJ g^–1^. Plant functional groups had a significant influence on LCV, with trees > shrubs > herbs, conifers > broadleaved trees, and evergreens > deciduous trees. The different values of LCV represented the long-term evolution and adaptation of plant species to different environments. Unexpectedly, no apparent latitudinal trends of LCV at community level were observed, although LCV at the species level clearly decreased with increasing latitude. Use efficiency of LCV (CUE, gC KJ^–1^), defined as the ratio of GPP to total LCV at the community level, varied quadratic with latitude and was lower in the middle latitudes. Climate (temperature and precipitation) may explain 52.9% of the variation in spatial patterns of CUE, which was positively correlated with aridity. Our findings are the first large-scale report of the latitudinal patterns of LCV in forests at the species, plant functional group, and community levels and provide new insights into the relationship between LCV and ecosystem functions in forest communities.

## Introduction

Leaf caloric value (LCV, KJ g^–1^) is an important plant trait that, to some extent, reflects the conversion efficiency of solar energy across plant leaves. LCV indicates how efficiently plants utilize sunlight, water, and other natural resources [1]. Like other plant functional traits, LCV has important influences on community structure and ecosystem functions, because it regulates the efficiency of energy use and its flow [2, 3].

The first report of LCV was a study on sunflowers [4]. Later studies [1, 5] compared the average LCV of dominant plant species among tropical rain forest, temperate forests, and alpine vegetation and concluded that LCV tended to increase with increasing altitude and latitude. Some studies demonstrated the variation of LCV over several species or over sites in a specific community [6]. Some researchers have explored the main factors that influence LCV and its relationship with seasonal fluctuations [7]. Other researchers have explored LCV as an adaptation mechanism for forest ecosystems and have tried to establish the relationship of LCV to productivity [8, 9]. In addition, several studies conducted the relative studies of LCV within single species or across several species within a single community.

However, few studies have investigated LCV in natural communities with regard to community composition, although community composition is always affected by convergent or divergent ecological strategies among species and plant functional group (PFG) [10]. According to Studing et al [11], plant functional traits may become amplified or dwarfed by different species assemblages, so changes in ecosystem processes cannot be predicted from the physiological or morphological traits of individual plants. Furthermore, it remains unclear whether the spatial patterns of LCV differ among species, PFG, and community on a large scale.

Scientists continue to grapple with the challenge of linking leaf traits to ecosystem functions in natural communities, although leaf traits are known to be important determinants of ecosystem functions [12, 13]. It is possible to aggregate functional traits measured from individual organisms to explain trends in populations, communities, and ecosystems [12–14]. Recently, one study reported that leaf stomatal densities at the community level were positively correlated with forest net primary productivity along the North-South Transect of Eastern China (NSTEC) [15]. Furthermore, net primary productivity, litter decomposition rate, and amounts of soil carbon and nitrogen were closely related to the leaf functional traits of communities [16]. On the basis of these findings, we proposed that LCV, a trait representing the conversion of solar energy across leaves, is closely related to gross primary productivity (GPP) at the community level. If that assumption is true, we further inferred that the use efficiency of LCV (CUE, gC KJ^–1^), defined as the ratio of GPP to total LCV at the community level, would appear as latitudinal patterns on a large scale (i.e., LCV pattern *vs*. CUE pattern).

We investigated the LCV across 745 common species in 9 typical forests along the NSTEC. Using these data, we explored the latitudinal patterns of LCV at the levels of species, PFG, and community and examined the possible association of LCV with forest productivity. Our main objectives were to 1) quantify LCV in forests at the species, PFG, and community levels on a large scale; 2) explore whether the LCV showed consistent latitudinal patterns within species, PFG, and community levels; and 3) establish the relationship of LCV to GPP and reveal the main factors that influence this relationship.

## Materials and Methods

### Site description

NSTEC is the 15^th^ standard transect of the International Geosphere-Biosphere Program. It is a unique forest belt that demonstrates a thermal gradient ranging from tropical rain forest to temperate coniferous forest. In this study, 9 natural forests along the NSTEC were selected for field sampling: Jianfengling (JF), Dinghu (DH), Jiulian (JL), Shennongjia (SN), Taiyue (TY), Dongling (DL), Changbai (CB), Liangshui (LS), and Huzhong (HZ). These places don’t required specific permissions, and free and open to the scientific research workers in China. We didn’t persecute any of endangered or protected species. These forests span latitudes from 18.7 to 51.8°N, with mean annual temperature (MAT) ranging from –4.4 to 20.9°C and mean annual precipitation (MAP) ranging from 481.6 to 2449.0 mm. Soils vary from tropical red soils with low organic matter to brown soils with high organic matter (Table 1). Forest vegetation varies correspondingly among tropical monsoon rainforest, subtropical evergreen forest, temperate deciduous forest, and cold-temperate coniferous forest. To minimize anthropogenic disturbances, we set up all sampling plots within well-protected national nature reserves for each forest type.

**Table 1:** Description of the study sites.

**Site**	**Latitude (°N)**	**Longitude (°E)**	**Altitude (m)**	**MAT (°C)**	**MAP (mm)**	**Vegetation type**
**JF**	18.74	108.86	809	19.80	2449.00	Tropical rain forest
**DH**	23.17	112.54	240	20.90	1927.00	Monsoon evergreen broad-leaved forest
**JL**	24.58	114.44	562	16.70	1954.00	Subtropical evergreen broad-leaved forest
**SN**	31.32	110.50	1510	10.60	1330.00	Mixed evergreen and deciduous broadleaved forest
**TY**	36.70	112.08	1668	6.20	662.00	Warm temperate deciduous broadleaved forest
**DL**	39.96	115.42	972	4.80	539.07	Warm temperate deciduous broadleaved forest
**CB**	42.40	128.09	758	2.60	691.00	Broad-leaved Korean pine forest
**LS**	47.19	128.90	401	-0.3	676.00	Broad-leaved Korean pine forest
**HZ**	51.78	123.02	850	-4.40	481.60	Temperate coniferous forest

HZ, Huzhong; LS, Liangshui; CB, Changbai; DL, Dongling; TY, Taiyue; SN, Shennongjia; JL, Jiulian; DH, Dinghu; JF, Jianfengling.

MAT, mean annual temperature; MAP, mean annual precipitation.

### Field sampling

The field survey was conducted between July and August of 2013. Four experimental plots (30 × 40 m) were selected in each forest ecosystem. The geographic details (latitude, longitude, and altitude), plant species composition, and community structure were determined for each plot. Trees were measured within the 30 × 40 m plots. Next, four plots (5 × 5 m) of shrubs and four plots (1 × 1 m) of herbs were chosen from the four corners of each (30 × 40 m) experimental plot. All common species were identified in each plot, including trees, shrubs, and herbs. The number, height, and diameter at breast height (DBH) of all trees for which this value ≥ 2 cm; basal stem diameter for shrubs; and aboveground live biomass of all herbs were measured. Subsequently, leaf samples were collected within the plot. We chose mature, healthy trees and collected fully expanded, sun-exposed leaves from four individuals of each plant species, using a long chainsaw or the services of two professional climbers. All of the leaf samples for a given plant species from one plot were mixed together as a single replicate [17]. In total, leaves of 745 plant species were collected (see Supplementary data of S1 File for each plant species). Species were divided by PFG (tree, shrub, or herb), leaf type (coniferous or broadleaved tree), and leaf type (evergreen or deciduous tree).

### Measurement of LCV

Leaf samples were cleaned and oven dried at 60°C to constant mass. Prior to measurement, the samples were ground by a high-speed mill and sieved through a 0.63-mm (or 40 mesh) standard screen to homogenize them. We measured the LCV (KJ g^–1^) of samples using the Parr 6300 automatic isoperibol calorimeter (Parr Instrument Company, Moline, IL, USA).

### Calculation and scaling-up of data

In order to consider the community composition of plant species and their relative leaf biomass in the specific community, we calculated LCV (KJ g^–1^) on three levels: 1) species level, averaging within species, 2) PFG level (Eq 1), weighted for the relative leaf biomass of the specific PFG (such as tree, shrub, or herb), and 3) community level (Eq 2), weighted for the relative leaf biomass of each species in the community [18].

$${\text{LCV}}_{\text{PFG}}=\sum_{i=1}^{n}P_{i}{\times \mathrm{LCV}}_{\text{i}}$$ (1)

$${\text{LCV}}_{\text{CWM}}=\sum_{j=1}^{m}P_{j}{\times \mathrm{LCV}}_{j}$$ (2)

where *P*_*i*_ is the relative leaf biomass of species *i* in the PFG (tree, shrub, or herb); *n* is the number of species in the PFG (tree, shrub, or herb); LCV_i_ is the LCV value of species *i*; LCV_PFG_ is the weighted mean of LCV at the PFG level, including tree (LCV_T_), shrub (LCV_S_), and herb (LCV_H_) values, respectively; *P*_*j*_ is the relative leaf biomass of species *j*; *m* is the number of species in the community; and, LCV_CWM_ is the weighted mean of LCV at the community level.

Leaf biomass for each tree species was calculated using species-specific allometric regressions on the measured values of DBH and height (H), which were obtained from the Chinese Ecosystem Research Net (CERN) database (http://159.226.111.42/pingtai/cernc/index.jsp; see details in Wang *et al*., 2015)[15]. Similarly, the leaf biomass of shrubs was calculated using the basal stem diameter and H through allometric approaches. For herbs, the leaf biomass was the oven-dried aboveground biomass. All the data on the GPP of each forest type were obtained from the MODIS products in a 1 × 1 km grid (http://daac.ornl.gov/cgi-bin/MODIS/GLBVIZ_1_Glb/modis_subset_order_global_col5.pl). MODIS GPP data from 2000 to 2010 for 9 sampling sites were selected. We used the average value of 10 years in this study[15].

### Use efficiency of LCV

GPP is the principal parameter by which leaf photosynthesis capacity is measured, and sunlight is the main energy source. Here, we defined the ratio of GPP to total LCV in a specific community as the use efficiency of LCV (CUE, gC KJ^–1^) (Eq 3).

$$\text{CUE}=\frac{\text{GPP}}{{\text{LCV}}_{\text{CWM}}{\times \text{B}}_{\text{Comm}}}$$ (3)

where B_Comm_ is the leaf biomass of community and LCV_CWM_ is the weighted mean of LCV at the community level (Eq 2).

### Climate data

The climate variables, including mean annual temperature (MAT), mean annual precipitation (MAP), maximum monthly temperature (T_max_), and maximum monthly precipitation (P_max_), were extracted from the climate dataset at a 1 × 1 km spatial resolution. The data were collected at 740 climate stations of the China Meteorological Administration, from 1961 to 2007, using the interpolation software ANUSPLIN [19].

The de Martonne aridity index (I) [20] was calculated to examine the effect of water availability:

$$\text{I}=\frac{\text{MAP}}{\text{MAT}+10}$$ (4)

### Statistical analysis

One-way ANOVA with the Least Significant Difference test was used to determine the statistical differences among LCV from different PFGs and communities. Regression analyses were conducted to test the latitudinal patterns of LCV at the species, PFG, and community levels and to describe the relationship between LCV and GPP at the community level. Redundancy analysis was used to identify the relative contribution of temperature and precipitation to the spatial patterns of CUE. All analyses were conducted using SPSS 13.0 (SPSS Inc., Chicago, IL, USA, 2004) and R software (version 2.15.2, R Development Core Team, 2012). All figures were produced by Sigmaplot 10.0 (Washington, IL, USA, 2006). The significance level was set at *p* = 0.05.

## Results

### Statistics for LCV

**Fig 1:**
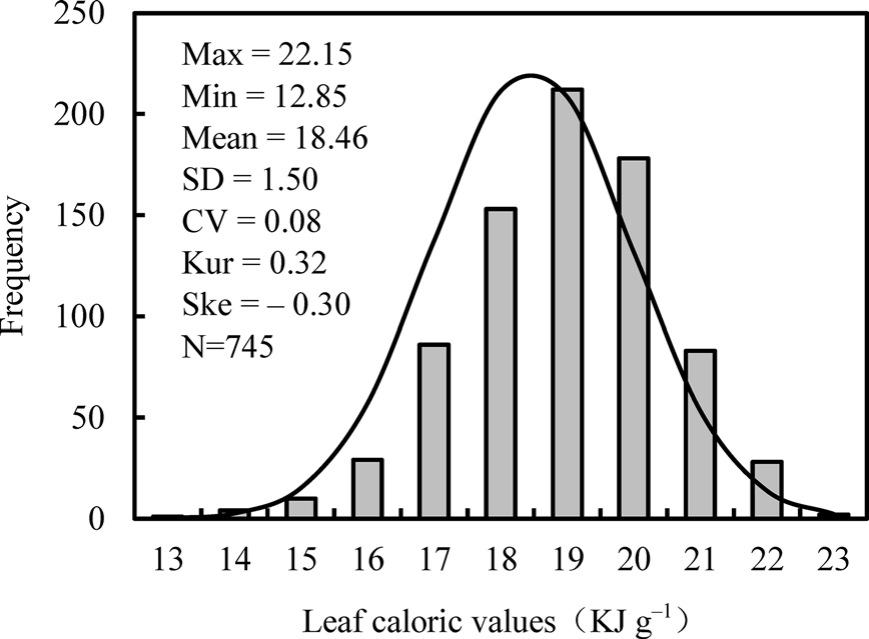
Frequency distribution of leaf caloric values in Chinese forests.

**Fig 2:**
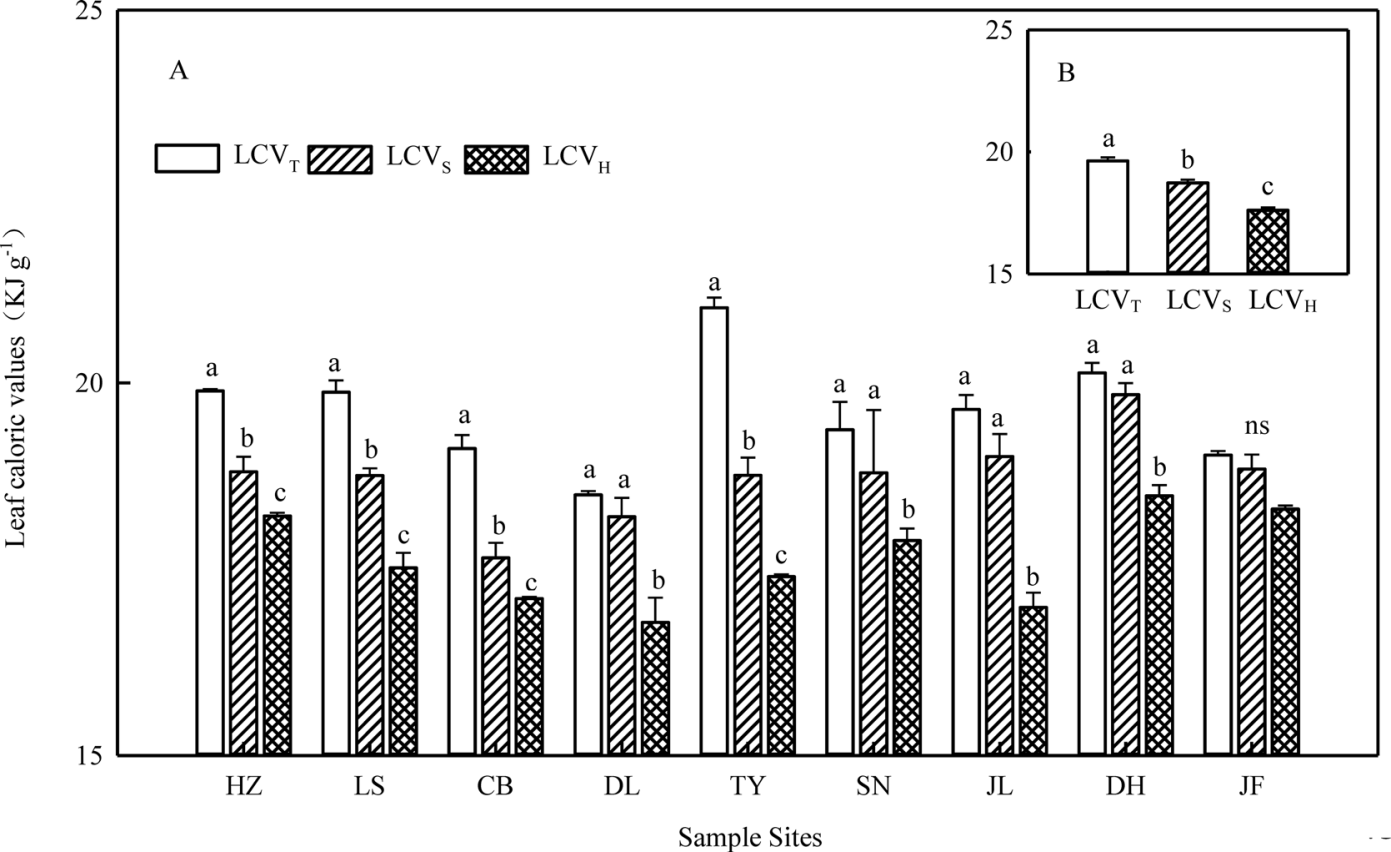
Changes in leaf caloric value among different plant functional groups. LCV_T_, LCVs, and LCV_H_, are the average of leaf caloric values weighted for the relative leaf biomass of trees, shrubs, and herbs, respectively. LCV_T_, LCVs, and LCV_H_, the average of leaf caloric values weighted for the relative leaf biomass of trees, shrubs, and herbs, respectively. HZ, Huzhong; LS, Liangshui; CB, Changbai; DL, Dongling; TY, Taiyue; SN, Shennongjia; JL, Jiulian; DH, Dinghu; JF, Jianfengling. Panel A and B were calculated at each site and for the total transect, respectively. Data are represented as mean ± S.E. Different letters indicate a significant difference among plant functional types (*p* < 0.05).

**Fig 3:**
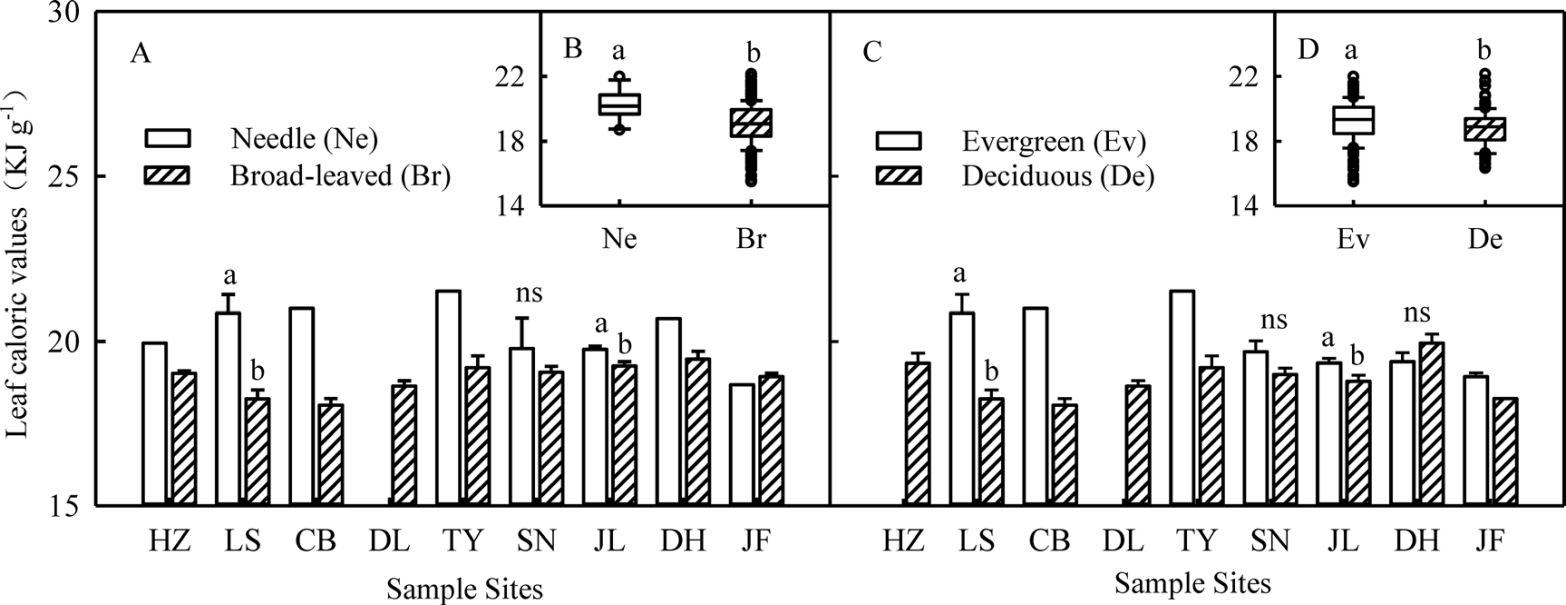
Changes in leaf caloric value among different types of trees in Chinese forests. Panel A, C and B, D were calculated at each site and for the total transect, respectively. Data are represented as mean ± S.E. Different letters indicate a significant difference among plant functional types (*p* < 0.05).

LCV at the species level showed a unimodal distribution in each type of forest (S1 Fig) and across the transect (Fig 1). Across all 745 species, LCV of species ranged from 12.85 to 22.15 kJ g^–1^ with an average of 18.46 KJ g^–1^ (Fig 1). Furthermore, LCV varied markedly across different PFGs (df = 2, F = 60.52, *p* < 0.001): LCV_T_ (19.64 KJ g^–1^) > LCV_S_ (18.73 KJ g^–1^) > LCV_H_ (17.61 KJ g^–1^) (Fig 2). Among trees, LCV was higher in conifers (20.25 KJ g^–1^) than in broadleaved trees (19.02 KJ g^–1^) and higher in evergreens (19.20 KJ g^–1^) than in their deciduous counterparts (18.01 KJ/g) (Fig 3). At the community level, LCV_CWM_ was significantly different among the 9 forests (*p* < 0.05), ranging from 18.33 KJ g^–1^ in DL to 20.20 KJ g^–1^ in TY (S1 Table).

### Latitudinal patterns of LCV and influencing factors

**Fig 4:**
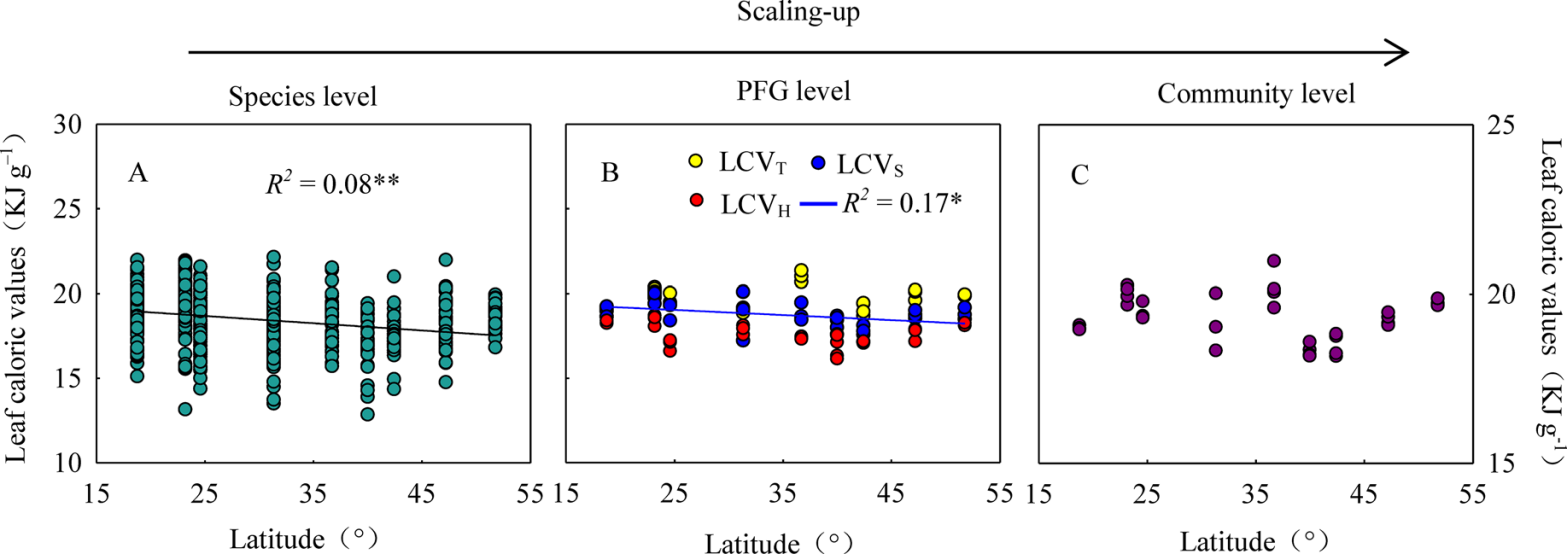
Latitudinal trends of leaf caloric values at the species, plant functional group (PFG), and community levels in Chinese forests. LCV_T_, LCVs, and LCV_H_, the average of leaf caloric value for the relative leaf biomass of trees, shrubs, and herbs, respectively. Only significant regression (*p* < 0.05) is given. *, *p* < 0.05; **, *p* < 0.01.

At the species level, LCV linearly decreased with increasing latitudes (*R*^*2*^ = 0.08, *p* < 0.001, Fig 4A). At the PFG level, LCV_S_ decreased clearly with increasing latitude (*R*^*2*^ = 0.17, *p* = 0.019), but similar trends were not observed for LCV_T_ or LCV_H_ (Fig 4B). Scaling up to the community level, LCV_CWM_ showed no significant latitude pattern (Fig 4C).

LCV was significantly correlated with climate factors (MAT, MAP, T_max_, and P_max_) at the species level. At the PFG level, LCV_T_ was not significantly correlated with climate factors, but LCV_S_ was positively correlated with MAT, MAP, T_max_, and P_max_. Unexpectedly, LCV_CWM_ was not significantly correlated with climate factors (Table 2).

**Table 2:** Pearson’s correlation between leaf caloric values and climate variables at species, plant functional groups (PFG), and community levels.

Level		N	Temperature		Precipitation	
			T_max_	MAT	P_max_	MAP
Species	LCV_i_	745	0.275**	0.270**	0.263**	0.296**
PFG	LCV_T_	345	–0.081	0.005	–0.06	–0.05
	LCV_S_	176	0.463**	0.467**	0.457**	0.455**
	LCV_H_	224	0.329	0.342	0.356*	0.450**
Community	LCV_CWM_	32	0.097	0.165	0.109	0.131

LCV_i_, the average of leaf caloric value for all plant species. LCV_T_, LCVs, and LCV_H_, the average of leaf caloric value for the relative leaf biomass of trees, shrubs, and herbs, respectively. LCV_CWM_, the average of leaf caloric value at the community level weighted by the relative biomass.

MAT, mean annual temperature; T_max_, maximum monthly temperature; MAP, mean annual precipitation; P_max_, maximum monthly precipitation

*, *p* < 0.05

**, *p* < 0.01.

### Changes in CUE along the transect

**Fig 5:**
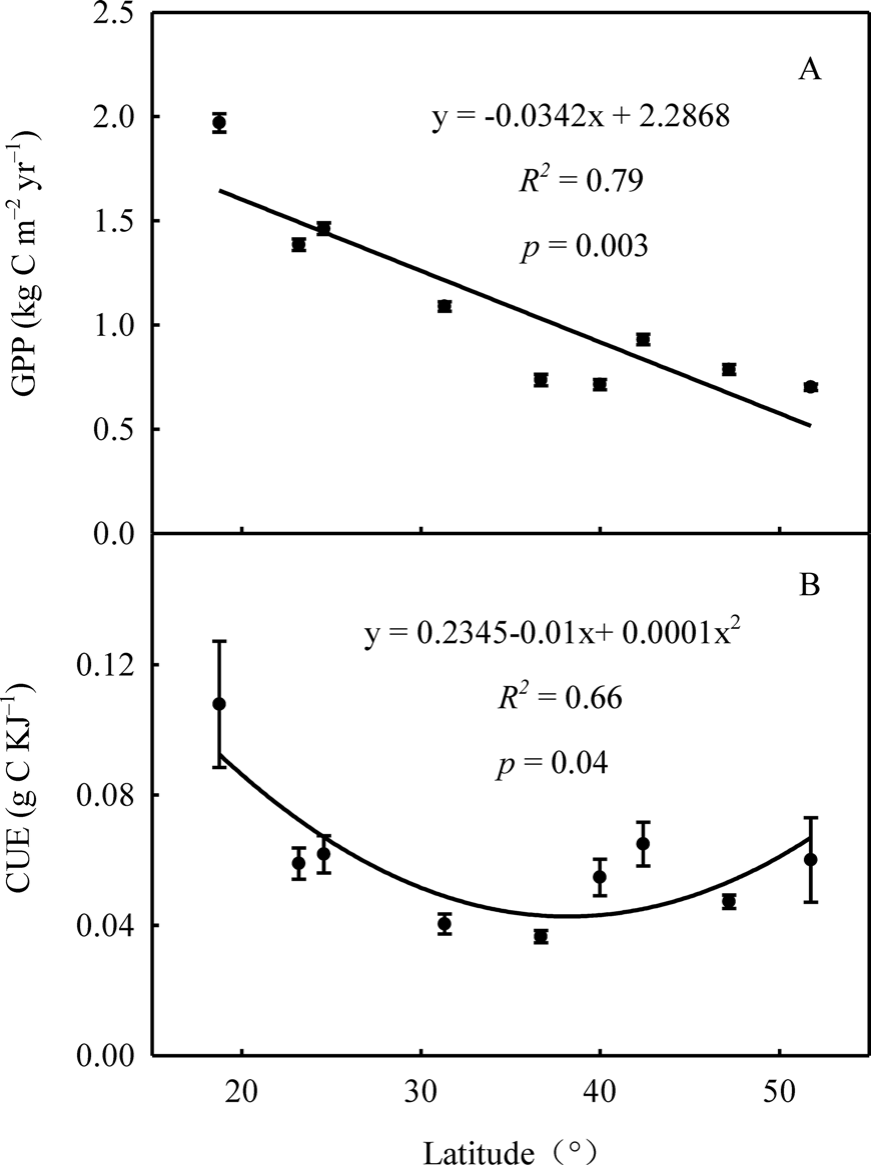
Latitudinal trends of gross primary productivity (GPP, kgC m^–2^ yr^–1^) and use efficiency of caloric value (CUE, gC KJ^–1^) at the community level. Data are represented as mean ± S.E.

LCV_CWM_ was not significantly correlated with GPP (S2 Fig) and forest leaf biomass along the transect. Interestingly, CUE showed a quadratic function with latitude (*R*^*2*^ = 0.65, *p* = 0.04); CUE varied from 0.037 to 0.108 gC KJ^–1^ and was lower in the middle latitudes (Fig 5). Among the 9 forests, CUE values significantly differed (*p* < 0.05); they were highest in JF and lowest in TY (S3 Fig).

**Fig 6:**
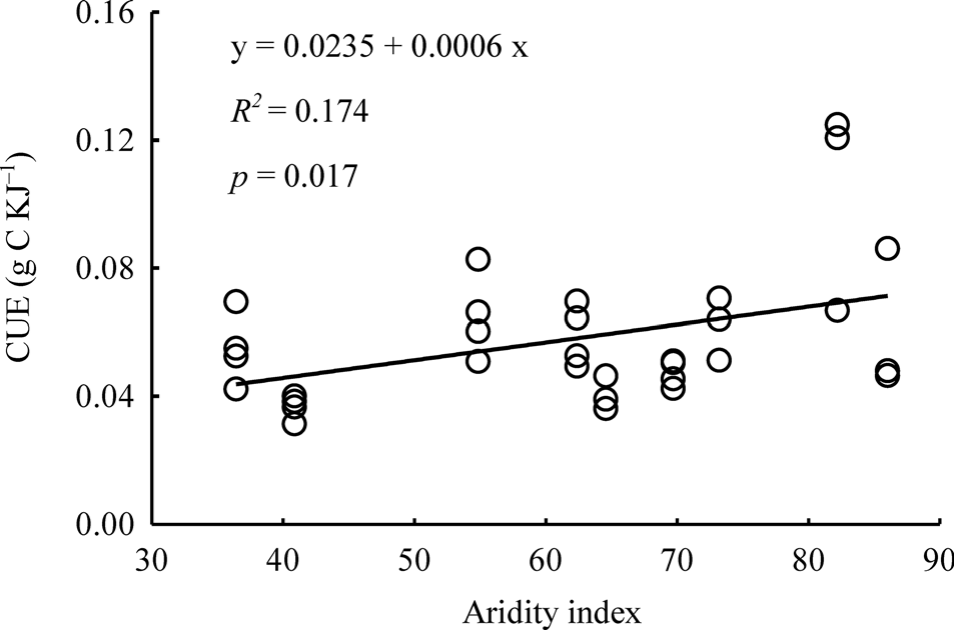
The linear relationship between use efficiency of caloric value (CUE) and aridity index.

CUE was significantly associated with climate factors (S2 Table). Redundancy analysis showed that temperature (MAT and T_max_) and precipitation (MAP and P_max_) might explain 52.9% of the variation in the latitudinal patterns of CUE (S4 Fig). Temperature alone may explain 8%, precipitation alone may account for 28%, and their interactions may explain 16.9% of variation. As an integrative effect, CUE values showed a significant linear correlation with the aridity index (*R*^*2*^ = 0.174, *p* = 0.017, Fig 6).

## Discussion

### Influence of plant functional groups and leaf forms on LCV

In Chinese forests, the mean LCV at the species level across all 745 species was approximately 18.46 KJ g^–1^. These values were consistent with the mean LCV for terrestrial plants worldwide (17.79 KJ g^–1^) [21]. Minor differences may result from inconsistencies in the leaf organ, forest age, phenology, leaf location, and other factors [22, 23]. In the present study, we collected leaf samples from mature natural forests (or long-term protected forests) in order to minimize the influence of stand age. Field sampling was conducted during the peak growth period of plants to minimize seasonal effects. Undeniably, careful attempts to perform representative sampling cannot entirely eliminate the effects of phenology along the 4700-km transect; some experiments have demonstrated that seasonal variation and phenology influence LCV [24, 25]. However, it is very difficult to perform repeated sampling over different seasons at a large scale.

Some previous studies also reported our same results that LCV_T_ > LCV_S_ > LCV_H_ [1, 26]. Long (1934) [4] measured the LCV of 12 cultivated species in different light gradients and reported that, LCV was positively correlated with light intensity. In natural forest communities, plant species, belonging to specific PFG, experience different light intensity, with trees receiving the most light, followed by shrubs and then herbs. Trees in the canopy layer can capture the most solar energy and accumulate higher amounts of energy through photosynthesis. Shrubs and herbs grown under shade conditions (or with insufficient sunlight) have a lower capacity to accumulate energy. In addition, most herbs are short-lived annual plants that may invest most of their energy in reproduction rather than in growth. Therefore, LCV decreased along a vertical gradient in forests, from the top to the bottom, reflecting the adaptation strategies of plant species to varying sunlight intensity. The LCV significantly differed among different PFGs, indicating the long-term evolution and adaptation of plant species to different environments.

The LCV values of different organic compounds in leaves were, in order, lipid (39.54 KJ g^–1^), resin (35.28 KJ g^–1^), protein (23.94 KJ g^–1^), carbohydrate (17.15 KJ g^–1^), and cellulose (16.8 KJ g^–1^) [27]. The leaves of coniferous trees contain more high-energy substances such as lipid and resin, which may be why higher LCVs were observed in this group. On the other hand, deciduous trees drop their leaves in the fall to reduce the loss of heat and evaporation in the winter. Deciduous trees also increase their carbon reserves in shoots during the short growing season, as an adaptation to low temperatures in winter [28]. Therefore, more energy fixed by the leaves of deciduous trees was transferred to the branches and stems in the summer. In contrast, higher LCV in evergreens may be explained by the fact that they must retain more energy in their leaves to satisfy caloric demand in their leaves in winter, compared with deciduous species that drop their leaves and so transfer more energy to other organs. Changes in LCV among different types of plants also indicated different adaptation strategies to environmental change at a large scale, as shown by previous studies in a single forest community or small region [29, 30].

### Different latitudinal patterns of LCV at different levels

LCV at the species level decreased with increasing latitude in the forest transect. A previous study investigated LCV of *Kandelia candel* in 8 sites form 19° 54' to 27° 51' and found that LCV decreased with increasing latitude in summer but increased in winter [31], findings that were verified by later work [32]. Another study investigated the LCV of dominant plants in four types of tropical forests in Panama, compared them with other published data, and concluded that tropical vegetation has lower LCV than do temperate or alpine vegetation; in other words, there was a gradient in the energy content of vegetation from the equatorial region to higher latitudes and altitudes [1, 5]. In addition, the LCV of polar tundra plants increased as the latitude increased [25]. The inconsistency in these results may be attributed to differences in the statistical analyses, due to consideration of all plant species in a specific community in this study vs. consideration only of some dominant species in Golley [1, 5]. Furthermore, the observed differences in latitudinal pattern between our study and these others may arise from differences in data sources. While the other referenced studies presented meta-analyses of published data, our study used a consistent sampling procedure that allowed us to control variables such as leaf organ, forest age, phenology, leaf location, and so on.

Unexpectedly, we found a latitudinal pattern at the species level, but not at the community level (LCV_CWM_), weighted according to the relative leaf biomass of each plant species. Therefore, the traditional average of dominant species or several species cannot reflect spatial patterns well without considering community composition. Grime’s mass ratio hypothesis states that community traits depend on species traits and the relative biomass contribution of each species in the community [33]. The relative importance of a particular species not only reflects the contribution of that species to the community but also represents the adaptation of the natural community to the environment. Previous studies also suggested that the ecological responses of plant traits at the species and community levels differed [34, 35]. Therefore, one must be careful about using species level data from dominant species to draw conclusions about large-scale patterns.

### Linkage of LCV to ecosystem functions

At the community level, the LCV_CWM_ and GPP were not directly correlated along the NSTEC. This result may be due to the impact of the climate of local small regions or the limitation of redundancy in species and community [36]. However, CUE (gC KJ^–1^) varied quadratic with latitude and was lower in the middle latitudes. This finding indicated that there was a close connection between LCV and ecosystem productivity through the regulation of CUE (not LCV_CWM_). The finding may result from the absence of a significant linear correlation between LCV_CWM_ and GPP. Some studies have provided additional evidence on the relationships between plant traits and ecosystem functions. Leaf size and leaf area index were closely correlated with ecosystem productivity [12, 13]. Leaf size and leaf area index were positively correlated with ecosystem productivity in three grasslands in Europe [37]. In addition, the spatial variation of stomatal density, at the community level, was closely related to ecosystem primary productivity [15]. Undoubtedly, linking the traits of individual plants or plant organs to ecosystem functions remains a major challenge for ecologists [13], but it is very important to understand the variation of ecosystem functions on a large scale and to predict the responses of terrestrial ecosystems to global change.

The latitudinal pattern of CUE was by temperature and precipitation, the two most important climate variables affecting vegetation distribution and plant traits on a large scale [38]. The significant positive correlation between CUE and the aridity index (Fig 6) also supports the idea that climate determines CUE. Leaf dry matter content is one of the most important parameters that reflect the adaptation of plant species to aridity on a global scale, and dry matter content is positively correlated with the aridity index [19]. However, leaves with higher dry matter content were likely to have higher CUE. In the middle latitudes of our study, MAP was lower and did not support optimal plant growth. In these arid regions, water deficiency not only limits GPP and photosynthesis but also alters plant species composition. The dominant species, including *Pinus tabuliformis* and *Biota orientalis*, have higher tolerance to drought due to their incrassate cuticle; greater stores of energy substances, such as lipid and protein; lower specific leaf area; and higher leaf dry matter content. In summary, our work provides evidence that the observed correlation between climate and CUE is being driven by changes in community composition and local adaptation of dominant plants to environmental conditions.

## Conclusions

The LCV of species in Chinese forests ranged from 12.85 to 22.15 KJ g^–1^, with an average of 18.46 KJ g^–1^. PFGs have an important influence on LCV, irrespective of site or transect scales, and the LCV declined in the order of trees > shrubs > herbs. Furthermore, LCV was higher in conifers than in broadleaved trees, and it was higher in evergreen than in deciduous trees. LCV in natural forest communities (LCV_CWM_), weighted for the relative leaf biomass of each plant species, shows no apparent latitudinal patterns from tropical forest to cold-temperate forest, although LCV at the species level decreased as latitude increased. Furthermore, CUE varied quadratic with latitude and was lower in the middle latitudes, an effect that was controlled by climate. Changes in LCV among different PFGs, tree types, and forest types represent the long-term evolution and adaptation of plant species to different environments.

## Supporting Information

S1 Fig Frequency distribution of leaf caloric values at nine sites. HZ, Huzhong; LS, Liangshui; CB, Changbai; DL, Dongling; TY, Taiyue; SN, Shennongjia; JL, Jiulian; DH, Dinghu; JF, Jianfengling.
(TIF)

S2 Fig The relationship between gross primary productivity (GPP, kgC m^–2^ yr^–1^) and leaf caloric value at the community level (LCV_CWM_, KJ g^-1^). (TIF)

S3 Fig Changes in use efficiency of leaf caloric value (CUE) in nine forests. Data were represented mean ± S.E. Different letters showed the significant difference among different forests (*p* < 0.05).
(TIF)

S4 Fig Variation partitioning (R^2^, %) of temperature and precipitation in accounting for the use efficiency of caloric value (CUE) at the community level. The letters *a* and *b* represented the independent effects of temperature and precipitation, respectively; ab represented the joint effect of temperature and precipitation.
(TIF)

S1 File Data of leaf caloric value in nine forest ecosystems in China. HZ, Huzhong; LS, Liangshui; CB, Changbai; DL, Dongling; TY, Taiyue; SN, Shennongjia; JL, Jiulian; DH, Dinghu; JF, Jianfengling.
(XLSX)

S1 Table Statistics of leaf caloric value at species, plant functional group (PFG), and community level for nine sites. HZ, Huzhong; LS, Liangshui; CB, Changbai; DL, Dongling; TY, Taiyue; SN, Shennongjia; JL, Jiulian; DH, Dinghu; JF, Jianfengling. LCV_i_, the average of leaf calorific value for all plant species. LCV_T_, LCV_S_, and LCV_H_ were the average of leaf calorific value on the relative leaf biomass for trees, shrubs, and herbs, respectively. LCV_CWM_, the average of leaf calorific value at community level weighted on the relative biomass. n, species number. CV, coefficient of variation. Data are represented as mean ± S.E.
(DOCX)

S2 Table Correlation matrix for use efficiency of caloric value (CUE) and climate variables. MAT, mean annual temperature; MAP, mean annual precipitation; T_max_, maximum monthly temperature; P_max_, maximum monthly precipitation; *, p < 0.05; **, p < 0.01.
(DOCX)
