# Supplementary figures and images for: Leaf Caloric Value from Tropical to Cold-Temperate Forests: Latitudinal Patterns and Linkage to Productivity
Source: PLoS One. 2016 Jun 24;11(6):e0157935. doi: 10.1371/journal.pone.0157935 (PMC4920410; doi:10.1371/journal.pone.0157935)

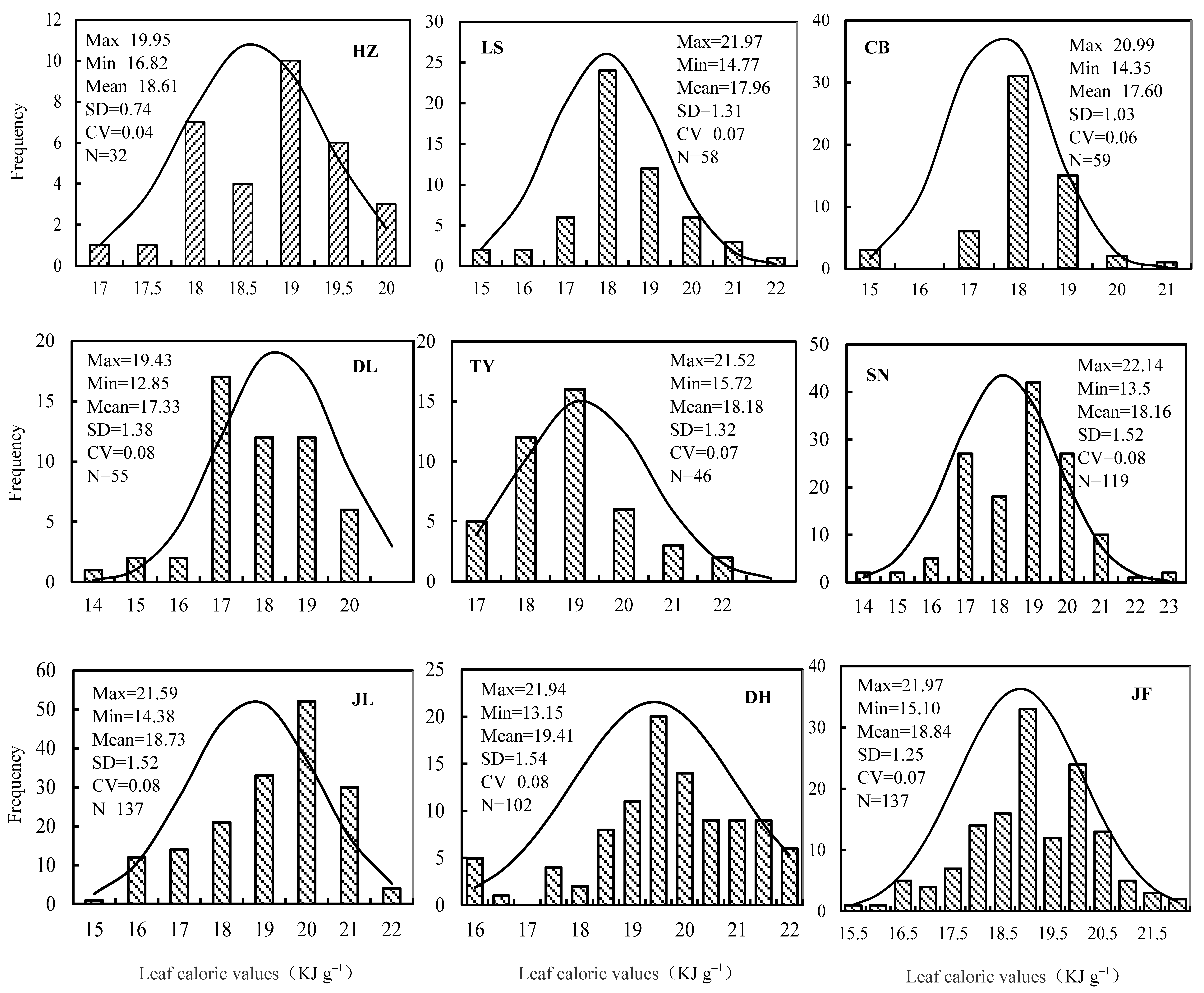

Supplement: S1 Fig — HZ, Huzhong; LS, Liangshui; CB, Changbai; DL, Dongling; TY, Taiyue; SN, Shennongjia; JL, Jiulian; DH, Dinghu; JF, Jianfengling. (TIF) [file pone.0157935.s001.tif]

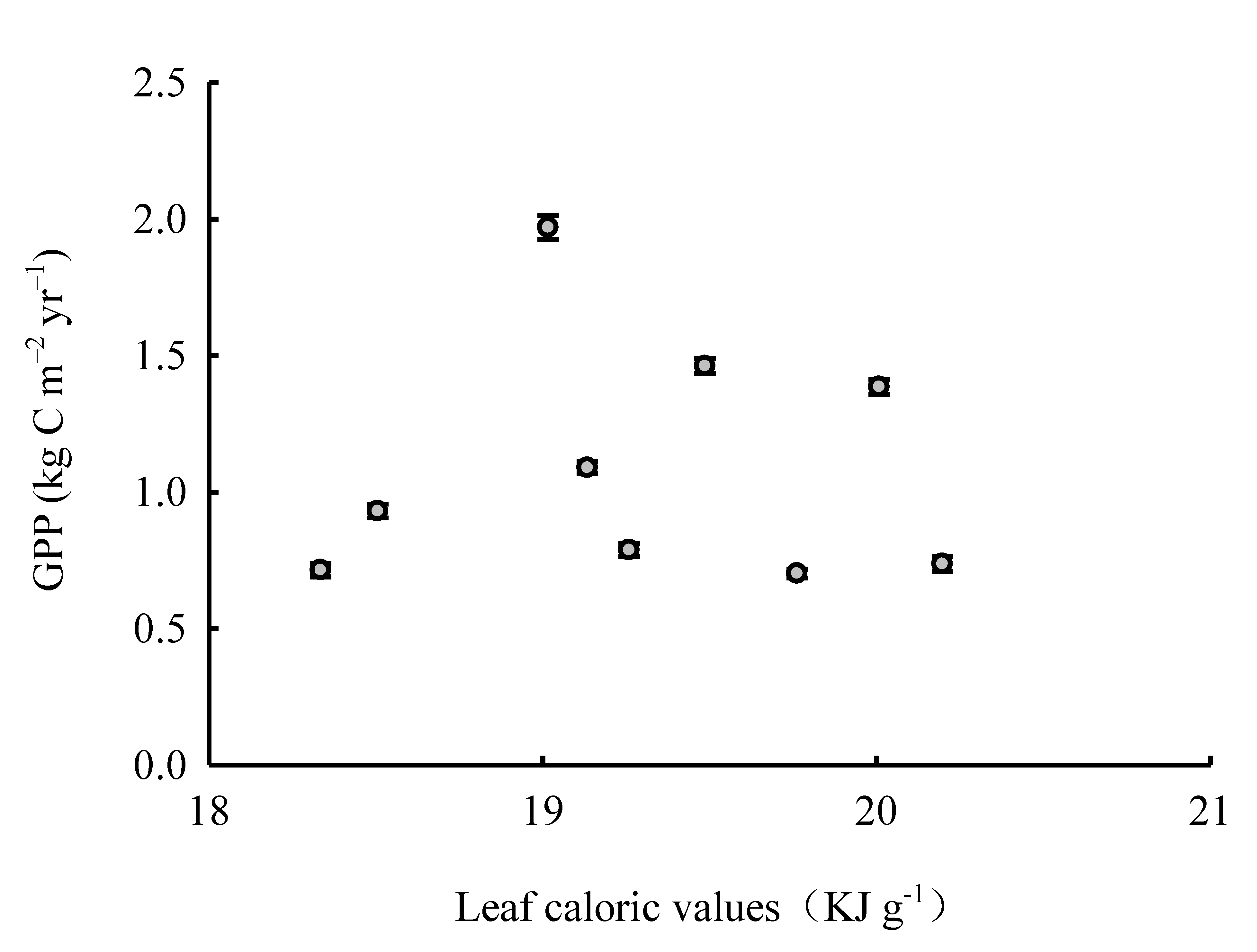

Supplement: S2 Fig — (TIF) [file pone.0157935.s002.tif]

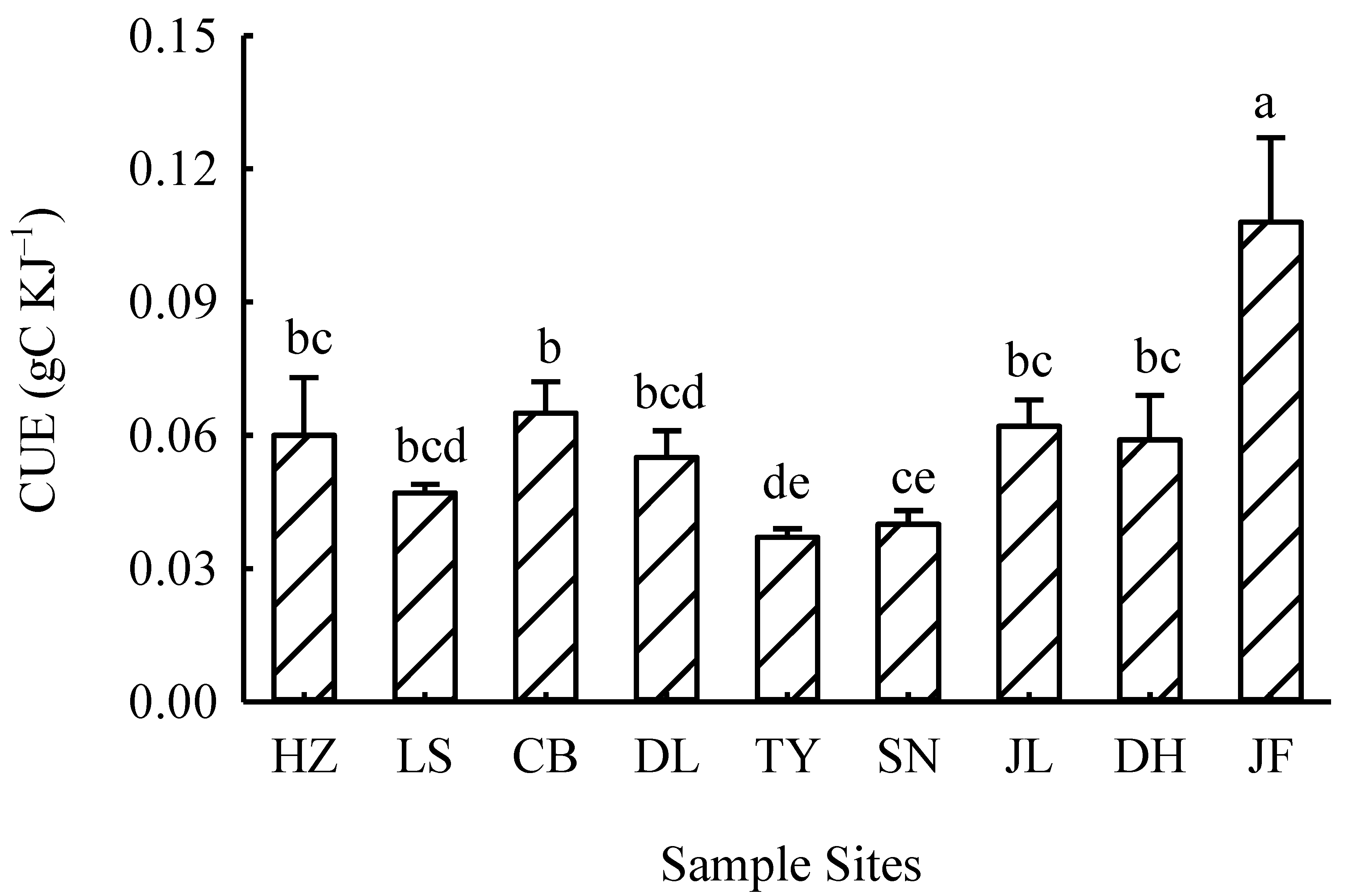

Supplement: S3 Fig — Data were represented mean ± S.E. Different letters showed the significant difference among different forests (p < 0.05). (TIF) [file pone.0157935.s003.tif]

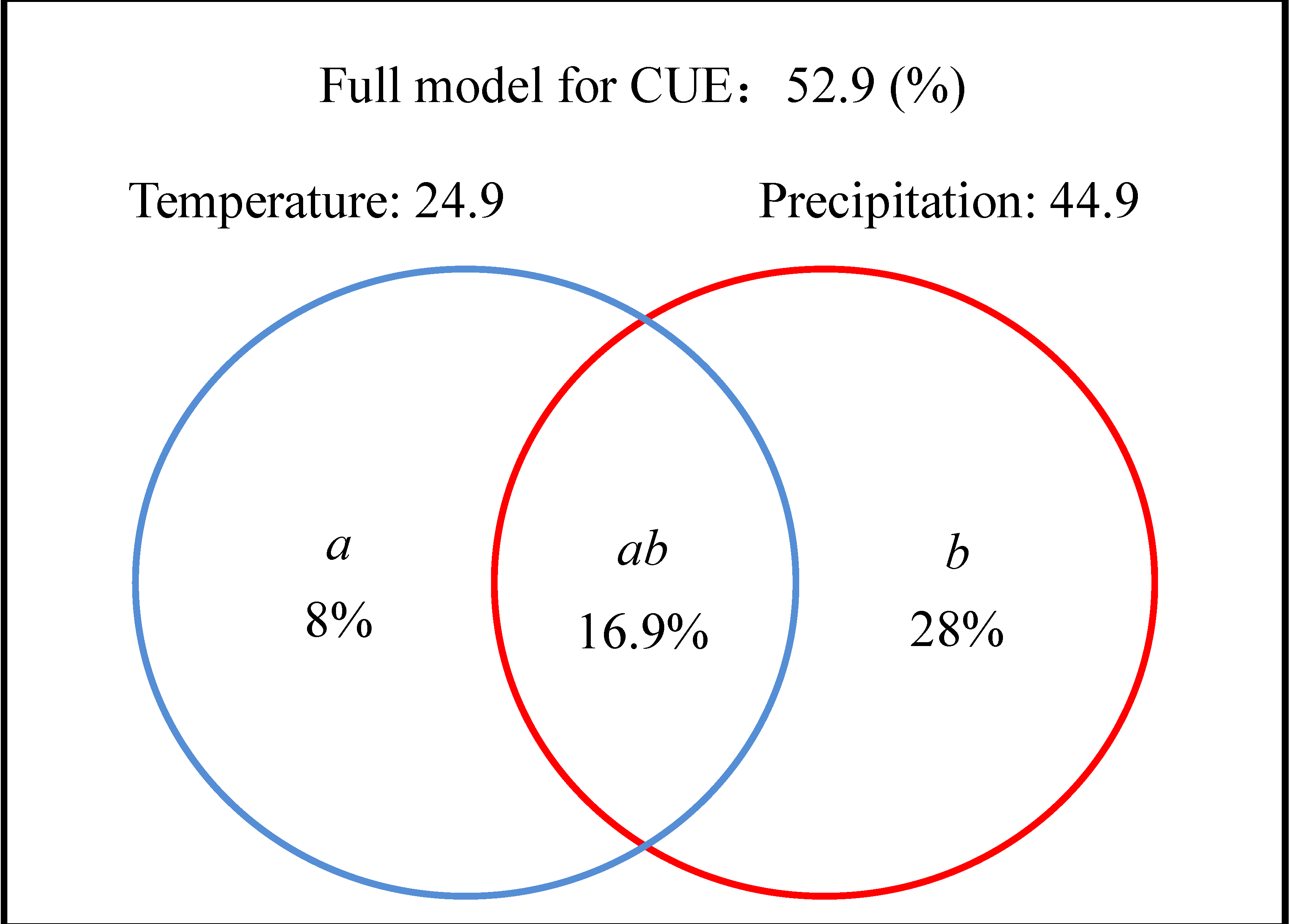

Supplement: S4 Fig — The letters a and b represented the independent effects of temperature and precipitation, respectively; ab represented the joint effect of temperature and precipitation. (TIF) [file pone.0157935.s004.tif]
