# Supplementary material for: Leaf Caloric Value from Tropical to Cold-Temperate Forests: Latitudinal Patterns and Linkage to Productivity
Source: PLoS One. 2016 Jun 24;11(6):e0157935. doi: 10.1371/journal.pone.0157935 (PMC4920410; doi:10.1371/journal.pone.0157935)
Supplement: S1 Table — HZ, Huzhong; LS, Liangshui; CB, Changbai; DL, Dongling; TY, Taiyue; SN, Shennongjia; JL, Jiulian; DH, Dinghu; JF, Jianfengling. LCVi, the average of leaf calorific value for all plant species. LCVT, LCVS, and LCVH were the average of leaf calorific value on the relative leaf biomass for trees, shrubs, and herbs, respectively. LCVCWM, the average of leaf calorific value at community level weighted on the relative biomass. n, species number. CV, coefficient of variation. Data are represented as mean ± S.E. (DOCX) [file pone.0157935.s006.docx]

**S1 Table. Statistics of leaf caloric value at species, plant functional group (PFG), and community level for nine forests**

| **Sits** | **Levels** | **Abbreviation** | **Mean ± SE** | **CV** | **No. of species** |
| --- | --- | --- | --- | --- | --- |
| HZ | Species | LCV_i_ | 18.61±0.13 | 0.039 | 32 |
|  | PFG | LCV_T_ | 19.89±0.02 | 0.002 | 3 |
|  |  | LCV_S_ | 18.81±0.21 | 0.019 | 8 |
|  |  | LCV_H_ | 18.22±0.04 | 0.004 | 21 |
|  | Community | LCV_CWM_ | 19.76±0.04 | 0.004 | 3 |
| LS | Species | LCV_i_ | 17.96±0.17 | 0.073 | 58 |
|  | PFG | LCV_T_ | 19.81±0.15 | 0.016 | 18 |
|  |  | LCV_S_ | 18.76±0.09 | 0.01 | 8 |
|  |  | LCV_H_ | 17.52±0.20 | 0.023 | 32 |
|  | Community | LCV_CWM_ | 19.26±0.07 | 0.008 | 4 |
| CB | Species | LCV_i_ | 17.60±0.14 | 0.059 | 59 |
|  | PFG | LCV_T_ | 18.68±0.18 | 0.019 | 18 |
|  |  | LCV_S_ | 17.65±0.20 | 0.023 | 11 |
|  |  | LCV_H_ | 17.11±0.02 | 0.003 | 30 |
|  | Community | LCV_CWM_ | 18.50±0.21 | 0.023 | 4 |
| DL | Species | LCV_i_ | 17.33±0.19 | 0.079 | 55 |
|  | PFG | LCV_T_ | 18.52±0.05 | 0.006 | 13 |
|  |  | LCV_S_ | 18.21±0.25 | 0.028 | 8 |
|  |  | LCV_H_ | 16.79±0.33 | 0.039 | 34 |
|  | Community | LCV_CWM_ | 18.33±0.09 | 0.01 | 4 |
| TY | Species | LCV_i_ | 18.18±0.19 | 0.073 | 46 |
|  | PFG | LCV_T_ | 20.84±0.14 | 0.013 | 13 |
|  |  | LCV_S_ | 18.76±0.24 | 0.025 | 16 |
|  |  | LCV_H_ | 17.40±0.03 | 0.004 | 17 |
|  | Community | LCV_CWM_ | 20.20±0.31 | 0.031 | 4 |
| SN | Species | LCV_i_ | 18.16±0.13 | 0.084 | 119 |
|  | PFG | LCV_T_ | 19.37±0.37 | 0.033 | 39 |
|  |  | LCV_S_ | 18.80±0.84 | 0.078 | 40 |
|  |  | LCV_H_ | 17.89±0.16 | 0.016 | 40 |
|  | Community | LCV_CWM_ | 19.13±0.48 | 0.044 | 3 |
| JL | Species | LCV_i_ | 18.73±0.12 | 0.081 | 137 |
|  | PFG | LCV_T_ | 19.65±0.19 | 0.017 | 81 |
|  |  | LCV_S_ | 19.01±0.30 | 0.027 | 35 |
|  |  | LCV_H_ | 16.99±0.20 | 0.02 | 21 |
|  | Community | LCV_CWM_ | 19.49±0.15 | 0.014 | 3 |
| DH | Species | LCV_i_ | 19.41±0.16 | 0.079 | 102 |
|  | PFG | LCV_T_ | 20.14±0.14 | 0.013 | 52 |
|  |  | LCV_S_ | 19.84±0.15 | 0.016 | 27 |
|  |  | LCV_H_ | 18.49±0.19 | 0.015 | 23 |
|  | Community | LCV_CWM_ | 20.01±0.12 | 0.013 | 4 |
| JF | Species | LCV_i_ | 18.84±0.11 | 0.067 | 137 |
|  | PFG | LCV_T_ | 19.04±0.05 | 0.005 | 108 |
|  |  | LCV_S_ | 18.85±0.19 | 0.018 | 23 |
|  |  | LCV_H_ | 18.31±0.05 | 0.004 | 6 |
|  | Community | LCV_CWM_ | 19.02±0.04 | 0.003 | 3 |

HZ, Huzhong; LS, Liangshui; CB, Changbai; DL, Dongling; TY, Taiyue; SN, Shennongjia; JL, Jiulian; DH, Dinghu; JF, Jianfengling.

LCV_i_, the average of leaf calorific value for all plant species. LCV_T_ , LCV_S_, and LCV_H_ were the average of leaf calorific value on the relative leaf biomass for trees, shrubs, and herbs, respectively. LCV_CWM_, the average of leaf calorific value at community level weighted on the relative biomass. n, species number. CV, coefficient of variation.

Data are represented as mean ± S.E.
