# Supplementary material for: Leaf Caloric Value from Tropical to Cold-Temperate Forests: Latitudinal Patterns and Linkage to Productivity
Source: PLoS One. 2016 Jun 24;11(6):e0157935. doi: 10.1371/journal.pone.0157935 (PMC4920410; doi:10.1371/journal.pone.0157935)
Supplement: S2 Table — MAT, mean annual temperature; MAP, mean annual precipitation; Tmax, maximum monthly temperature; Pmax, maximum monthly precipitation; *, p < 0.05; **, p < 0.01. (DOCX) [file pone.0157935.s007.docx]

**S2 Table. Correlation matrix for use efficiency of caloric value (CUE) and climate variables**

|  | CUE | MAT | T_max_ | MAP | P_max_ |
| --- | --- | --- | --- | --- | --- |
| CUE | 1 |  |  |  |  |
| MAT | 0.354* | 1 |  |  |  |
| T_max_ | 0.469** | 0.903** | 1 |  |  |
| MAP | 0.514** | 0.929** | 0.872** | 1 |  |
| P_max_ | 0.375* | 0.938** | 0.861** | 0.965** | 1 |

MAT, mean annual temperature; MAP, mean annual precipitation; T_max_, maximum monthly temperature; P_max_, maximum monthly precipitation;

*, *p* < 0.05; **, *p* < 0.01.
